# Differential expression of serum biomarkers in hemodialysis patients with mild cognitive decline: A prospective single-center cohort study

**DOI:** 10.1038/s41598-018-29760-5

**Published:** 2018-08-16

**Authors:** Bin Zhu, Li-Na Jin, Jian-Qin Shen, Jin-Feng Liu, Ri-Yue Jiang, Ling Yang, Jie Zhang, Ai-Lin Luo, Li-Ying Miao, Chun Yang

**Affiliations:** 1 Department of Critical Care Medicine, The Third Affiliated Hospital of Soochow University, Changzhou, China; 2 The Blood Purification Center, The Third Affiliated Hospital of Soochow University, Changzhou, China; 3 Department of Radiation Oncology, The Third Affiliated Hospital of Soochow University, Changzhou, China; 4 Department of Cardiology, The Third Affiliated Hospital of Soochow University, Changzhou, China; 5 Department of Anesthesiology, Tongji Hospital, Tongji Medical College, Huazhong University of Science and Technology, Wuhan, China

## Abstract

Studies suggest that hemodialysis patients are at a higher risk for cognitive decline than healthy individuals; however, underlying mechanisms have not been fully elucidated. We aimed to investigate the roles of serum biomarkers, such as brain-derived neurotrophic factor (BDNF), inflammatory cytokines, fibroblast growth factor (FGF)-23 and its co-receptor α-klotho and platelet (PLT) count in mild cognitive decline (MCD) of patients undergoing hemodialysis in this prospective cohort study. Serum levels of BDNF, tumour necrosis factor (TNF)-α, interleukin (IL)-6 and the number of PLT were significantly altered in the MCD group compared with those in healthy controls (HCs) or those with normal cognitive function (NCF). Although serum α-klotho and FGF-23 levels were significantly altered in the MCD group, there were no statistical differences between the MCD and NCF groups. Serum BDNF levels and PLT counts were significantly correlated with cognitive test scores. Receiver operating characteristic (ROC) curves demonstrated that BDNF and PLT were potential biomarkers for improved MCD diagnosis in patients with hemodialysis. These findings suggest that hemodialysis-related MCD is associated with altered BDNF, TNF-α and IL-6 levels as well as PLT counts and that serum BDNF levels and PLT counts are potential biomarkers for hemodialysis-related MCD diagnosis.

## Introduction

Chronic kidney disease (CKD) has increasingly become a global public health problem^1^. Epidemiological studies show that the incidence of CKD in adults is approximately 15% in developed countries^2,3^. In China, approximately 10.8% (120 million) people suffer from CKD^4^. Cognitive functioning encompasses an individual’s psychological understanding and processing and consists of multiple domains including memory, computing, time and space orientation, structural ability, executive functioning and understanding, expression and application of language^5^. Furthermore, the spectrum of cognitive decline, ranging from mild cognitive impairment to dementia, is clinically dependent on varying degrees of impairment caused by several undetermined pathological causes^6^. A growing body of evidence demonstrates that the incidence of neurological and psychiatric symptoms, such as depression, anxiety and altered cognitive function, is significantly higher in hemodialysis patients than in healthy individuals^7,8^. Therefore, a better understanding of the cognitive decline observed in patients with CKD undergoing hemodialysis is needed.

Cognitive decline occurs not only in the end-stage renal disease but also in earlier stages of CKD^9,10^. Although the pathological mechanisms of CKD-related cognitive dysfunction remain unclear, the adverse impact toxins that are not efficiently cleared from the circulation might be associated with altered cognitive function. Interestingly, we found that some non-elderly patients with normal cognitive function (NCF) before hemodialysis developed mild cognitive decline (MCD) after several years of hemodialysis. In addition, a cross-sectional cohort study including 108 maintenance hemodialysis patients reported that approximately 25% of individuals were cognitively declined^11^. These findings highlight the striking deleterious effects of hemodialysis on cognitive function of patients.

Hemodialysis is an independent risk factor for MCD and seriously affects the quality of life in patients with CKD, also leading to prolonged hospitalisation and increased risk of death^12,13^. However, the mechanisms underlying hemodialysis-related MCD have not yet been fully elucidated. A clinical study by Drew *et al*.^14^ included 263 hemodialysis patients indicated that serum fibroblast growth factor (FGF)-23 was highly associated with poor performance on a cognition test. Other than FGF-23, a limited number of studies investigated the role of other serum biomarkers in the pathogenesis of hemodialysis-related MCH. We therefore aimed to assess whether other serum biomarkers were involved in the pathogenesis of hemodialysis-related MCH. Specifically, we compared the levels of brain-derived neurotrophic factor (BDNF), α-klotho and its coreceptor FGF23, tumour necrosis factor (TNF)-α, interleukin (IL)-6 and platelet (PLT) number among hemodialysis patients with MCD or normal cognitive function (NCF) and healthy control (HC) subjects. We also evaluated correlations between cognition test scores and serum biomarkers and performed receiving operating characteristic (ROC) curves to assess whether certain serum biomarkers could predict and diagnose hemodialysis-related MCD.

## Results

### General clinical characteristics of patients receiving hemodialysis

Table 1 shows the summary of the clinical characteristics of the study patients including age, sex, routine blood test values and biomedical parameters. Briefly, haemoglobin, haematocrit, red blood cell count, blood urea nitrogen, creatinine and albumin were significantly different among the MCD, NCF and HC groups, whereas age, sex, white blood cell (WBC) count, dialysis period, serum calcium, serum phosphate, parathyroid hormone, dry body weight or education history were not different among the three groups.

**Table 1:** Clinical and biological parameters of patients among the three groups.

Variable	HC (n = 20)	NCF (n = 29)	MCD (n = 29)	F	t	χ^2^	*P*
**Male [n (%)]**	11 (55.00)	15 (51.70)	15 (51.70)			0.11	0.97
**Age (years)**	45.00 ± 8.90	43.10 ± 7.80	48.00 ± 7.20	2.87			0.06
**Education (years)**	11.10 ± 3.06	11.24 ± 2.46	10.83 ± 2.25	0.19			0.82
**Type of kidney diseases**						9.88	0.27
Chronic glomerulonephritis	—	17	15				
Diabetic nephropathy	—	1	5				
Benign arteriolar nephrosclerosis	—	0	1				
Polycystic kidney	—	1	1				
IgA nephropathy;	—	0	1				
Medullary sponge kidney	—	0	1				
Chronic kidney disease of unknown etiology	—	6	5				
Nephrotic syndrome	—	3	0				
Systemic lupus erythematosus nephropathy	—	1	0				
**HB (g/L)**	142.20 ± 20.20	107.90 ± 13.00	108.90 ± 12.80	37.23			<0.001***
**HCT (L/L)**	0.43 ± 0.05	0.33 ± 0.04	0.33 ± 0.04	38.54			<0.001***
**WBC (10** ^**9/L**^ **)**	6.14 ± 1.26	5.99 ± 1.80	5.79 ± 1.61	0.29			0.75
**RBC (10** ^**12/L**^ **)**	4.94 ± 0.45	3.69 ± 0.50	3.69 ± 0.52	47.15			<0.001***
**BUN (mmol/L)**	4.40 ± 0.85	27.55 ± 5.71	24.33 ± 4.42	179.30			<0.001***
**Cr (umol/L)**	71.55 ± 8.65	1052.00 ± 195.90	965.00 ± 172.50	258.40			<0.001***
**ALB (g/L)**	46.04 ± 2.49	40.04 ± 1.99	41.46 ± 4.03	24.40			<0.001***
**Dialysis duration (months)**	—	71.52 ± 38.13	78.07 ± 47.39		0.58		0.56
**Calcium (mmol/L)**	—	2.15 ± 0.24	2.25 ± 0.29		1.34		0.18
**Phosphate (mmol/L)**	—	2.25 ± 0.70	2.07 ± 0.69		0.96		0.33
**PTH (pg/mL)**	—	534.3 ± 478.2	420.5 ± 362.6		1.02		0.31
**Dry weight (kg)**	—	61.42 ± 11.25	58.00 ± 8.28		1.32		0.19

Data are shown as means ± standard error of means (n = 20–29), ***p < 0.001. ALB: albumin; BUN: blood urea nitrogen; Cr: creatinine; HB: haemoglobin; HC: healthy control group; HCT: haematocrit; MCD: mild cognitive decline group; NCF: normal cognitive function group; PLT: blood platelet; PTH: parathyroid hormone RBC: red blood cell; WBC: white blood cell.

### Cognitive function is worse in hemodialysis patients with MCD than those with NCF

**Figure 1:**
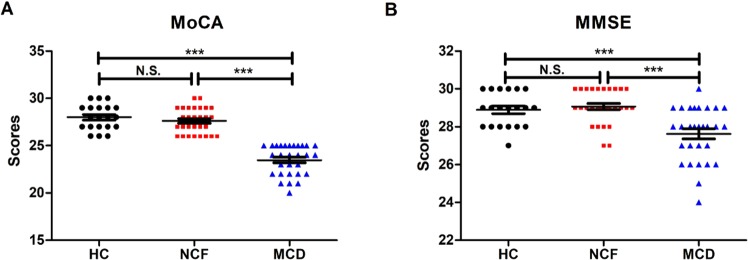
Assessment of cognitive function by MoCA and MMSE. (**A**) MoCA scores among the three study groups (F_2,75_ = 83.21_,_ *p* < 0.0001). (**B**) MMSE scores among the groups (F_2,75_ = 13.89_,_ *p* < 0.0001). Data are shown as means ± standard error of means (n = 20–29); ***p < 0.001. HC: healthy control; MCD: mild cognitive decline; MMSE: Mini-Mental State Examination; MoCA: Montreal Cognitive Assessment; NCF: normal cognitive function; N.S.: not significant.

CKD patients undergoing hemodialysis were divided based on their performance on two cognitive function tests: the Montreal Cognitive Assessment (MoCA) (Fig. 1A) and the Mini-Mental State Examination (MMSE) (Fig. 1B). We found that MoCA scores were significantly lower in the MCD group than in the HC and NCF groups, whereas no significant differences were observed between the HC and NCF groups. Similarly, MMSE also exhibited a significant decrease in the MCD group compared with the other two groups.

### Serum PLT count and biomarker levels

**Figure 2:**
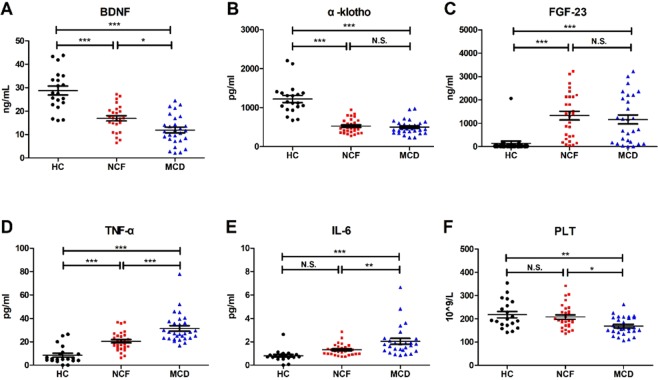
Serum levels of biomarkers among the three study groups. (**A**) Serum BDNF levels (F_2,70_ = 37.09_,_ *p* < 0.0001). (**B**) Serum α-klotho levels (F_2,75_ = 57.26_,_ *p* < 0.0001). (**C**) Serum FGF-23 levels (F_2,74_ = 11.70_,_ *p* < 0.0001). (**D**) Serum TNF-α levels (F_2,75_ = 32.37_,_ *p* < 0.0001). (**E**) Serum IL-6 levels (F_2,72_ = 11.80_,_ *p* < 0. 0001). (**F**) Blood PLT count (F_2,75_ = 7.04_,_ *p* < 0.001). Data are shown as means ± standard error of means (n = 5–7 or 20). *p < 0.05, **p < 0.01 or ***p < 0.001. BDNF: brain-derived neurotrophic factor; FGF-23: fibroblast growth factor-23; HC: healthy control; IL-6: interleukin-6; MCD: mild cognitive decline; NCF: normal cognitive function; N.S.: not significant; PLT: platelet; TNF-α: tumour necrosis factor-α.

Serum BDNF levels were significantly lower in both the MCD and NCF groups than the HC group. Intriguingly, there was a significant decrease in serum BDNF levels in the MCD group compared with the NCF group (Fig. 2A). FGF-23 and α-klotho levels were significantly different among the three groups. Specifically, serum α-klotho levels were significantly down-regulated in both the MCD and NCF groups, compared with the HC subjects (Fig. 2B). In contrast, serum FGF-23 levels were significantly increased in both the MCD and NCF groups compared with the HC group (Fig. 2C). However, serum α-klotho and FGF-23 levels failed to show a significant change between the MCD and NCF groups. In the present study, we also measured serum levels of several inflammatory cytokines. We found that both TNF-α and IL-6 levels were significantly higher in the MCD group than the HC group, whereas they were both significantly lower in the NCF group compared to the MCD group. Interestingly, when compared with the HC group, TNF-α but not IL-6 was higher in the NCF group (Fig. 2D,E). Finally, CKD patients with MCD exhibited significantly decreased PLT counts compared with the HC and the NCF groups (Fig. 2F).

### Correlation of MoCA score with serum biomarkers

**Figure 3:**
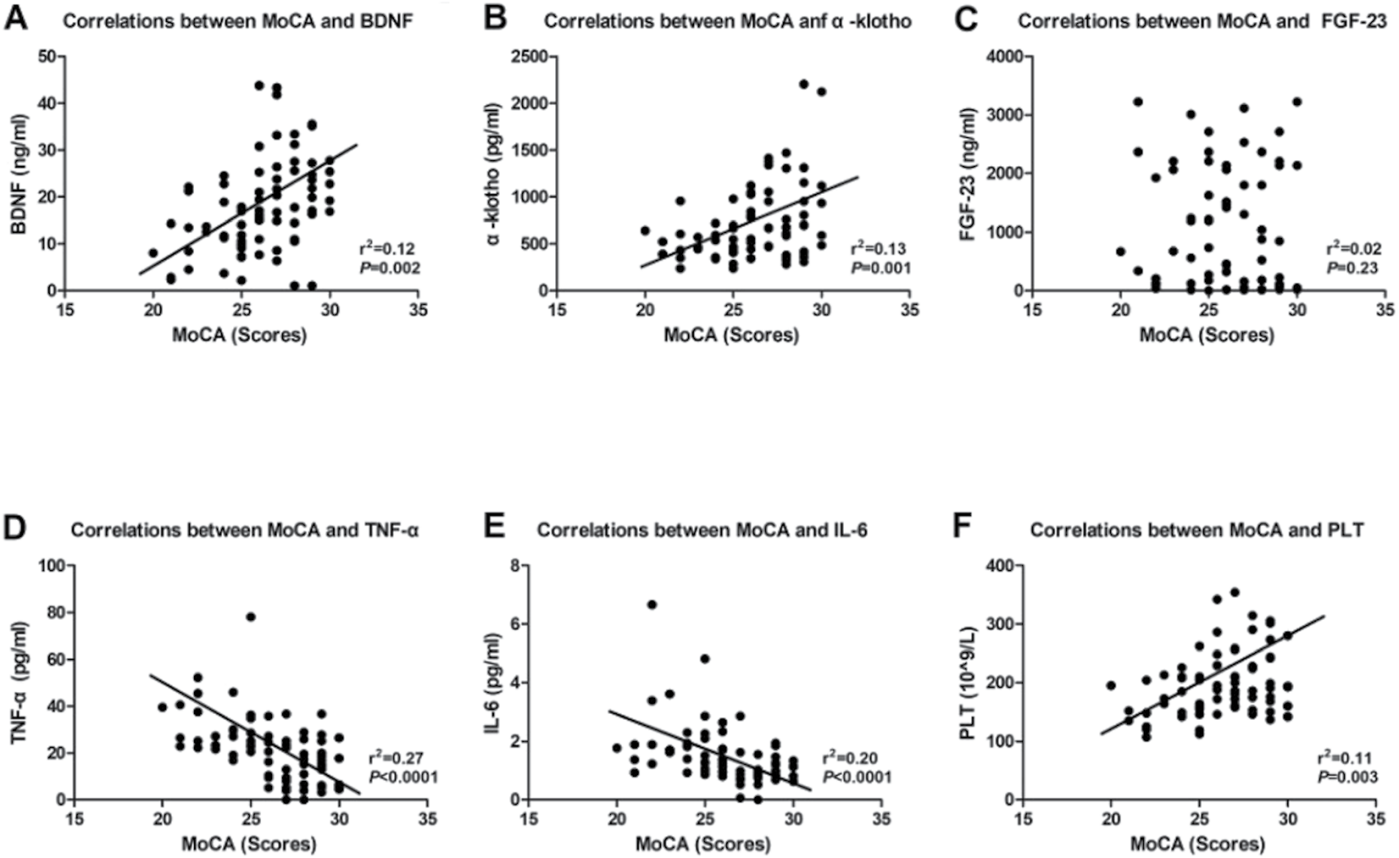
Correlations between MoCA and serum biomarkers. (**A**) Correlation between MoCA and serum BDNF (r^2^ = 0.12, *p* = 0.002). (**B**) Correlation between MoCA and serum α-klotho (r^2^ = 0.13, *p* = 0.002). (**C**) Correlation between MoCA and serum FGF-23 (r^2^ = 0.02, *p* = 0.23). (**D**) Correlation between MoCA and serum TNF-α (r^2^ = 0.27, *p* < 0.0001). (**E**) Correlation between MoCA and serum IL-6 (r^2^ = 0.02, *p* < 0.0001). (**F**) Correlation between MoCA and blood PLT count (r^2^ = 0.11, *p* = 0.003). BDNF: brain-derived neurotrophic factor; FGF-23: fibroblast growth factor-23; IL-6: interleukin-6; PLT: platelet; TNF-α: tumour necrosis factor-α. MoCA: Montreal Cognitive Assessment.

We next investigated the potential correlations among serum biomarkers and the MoCA scores. Our analysis revealed that BDNF, α-klotho and PLT count were positively correlated with the MoCA score (Figs 3A,B,F), whereas TNF-α and IL-6 were negatively correlated with the MoCA score (Fig. 3D,E). Interestingly, several lines of evidence suggest that FGF-23 plays a key role in the molecular mechanisms of CKD and hemodialysis as well as the associated cognitive dysfunction. However, in the present study, we failed to show a significant correlation between FGF-23 and the MoCA score (Fig. 3C).

### Correlation of MMSE score with serum biomarkers

**Figure 4:**
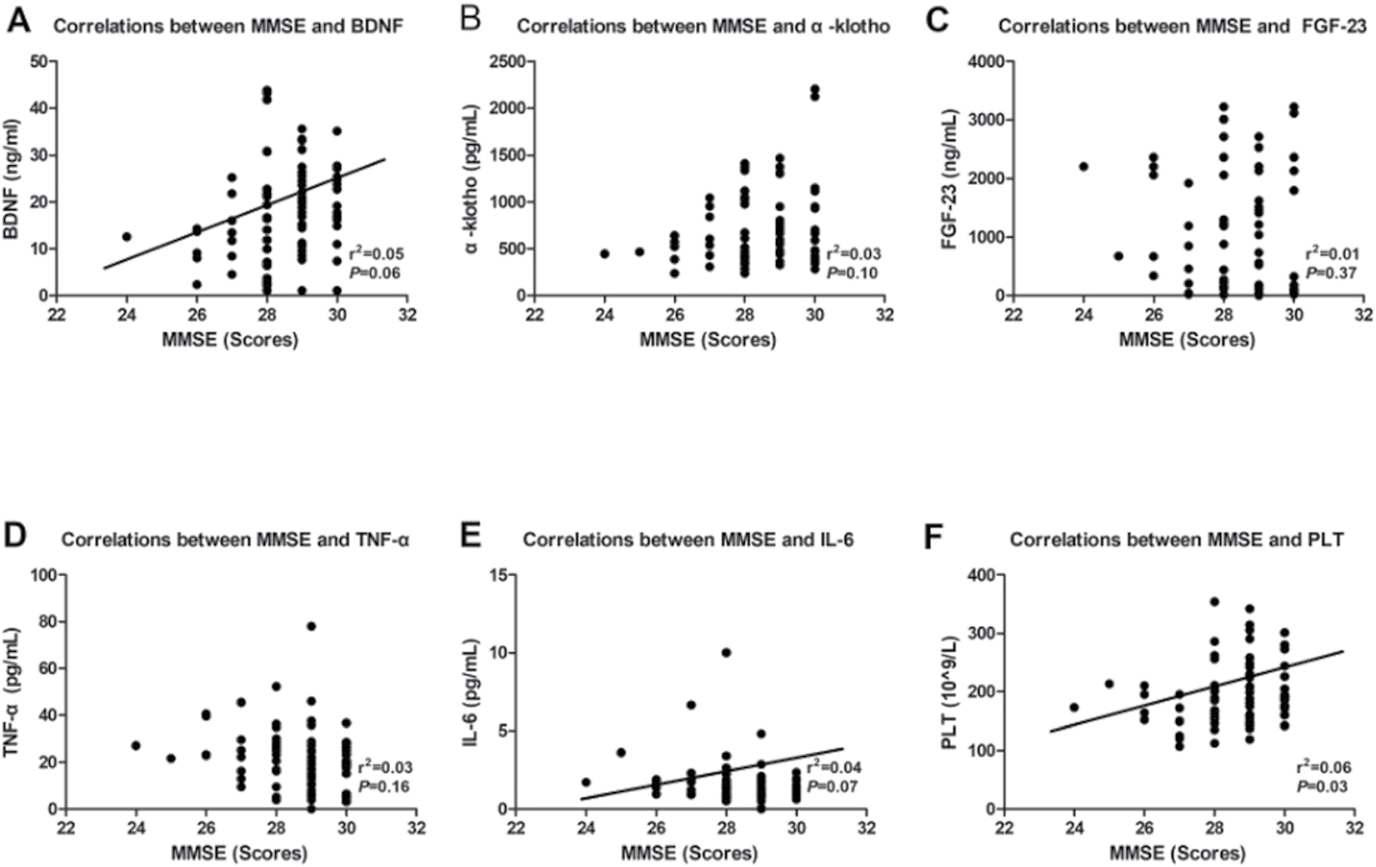
Correlations between MMSE and serum biomarkers. (**A**) Correlation between MMSE and serum BDNF (r^2^ = 0.05, *p* = 0.006). (**B**) Correlation between MMSE and serum α-klotho (r^2^ = 0.03, *p* = 0.10). (**C**) Correlation between MMSE and serum FGF-23 (r^2^ = 0.01, *p* = 0.37). (**D**) Correlation between MMSE and serum TNF-α (r^2^ = 0.03, *p* = 0.16). (**E**) Correlation between MMSE and serum IL-6 (r^2^ = 0.04, *p* = 0.07). (**F**) Correlation between MMSE and blood PLT count (r^2^ = 0.06, *p* = 0.003). BDNF: brain-derived neurotrophic factor; FGF-23: fibroblast growth factor-23; IL-6: interleukin-6; PLT: platelet; TNF-α: tumour necrosis factor-α; MMSE: Mini-Mental State Examination.

Our analysis also revealed that BDNF and PLT count were independently correlated with the MMSE score (Fig. 4A,F); however, other biomarkers including α-klotho, FGF-23, TNF- α and IL-6 did not correlate with the MMSE score (Fig 4B–E).

### Evaluation of serum biomarkers for the diagnosis of hemodialysis-related MCD using ROC curve analysis

**Figure 5:**
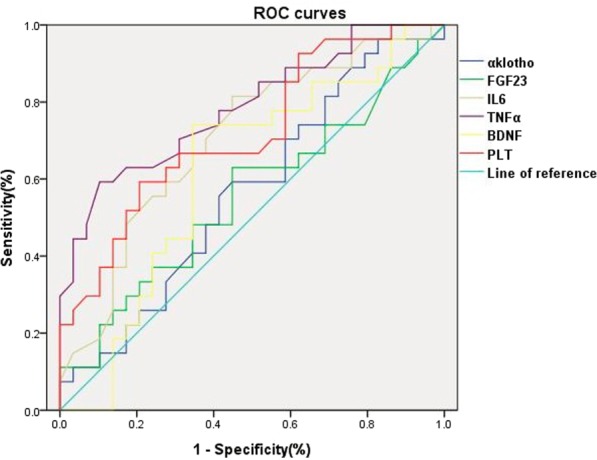
ROC curves of serum biomarkers including α-klotho, FGF-23, IL-6, TNF-α, BDNF and PLT count for the diagnosis of hemodialysis-related MCD. BDNF: brain-derived neurotrophic factor; FGF-23: fibroblast growth factor-23; IL-6: interleukin-6; MCD: mild cognitive decline; PLT: platelet; ROC: receiver operating characteristic; TNF-α: tumour necrosis factor-α.

The best cut-off values for serum α-klotho, FGF-23, IL-6, TNF-α, PLT count and BDNF levels for the diagnosis of dialysis-induced MCD were 469.5 pg/ml, 1264.5 ng/ml, 1.8 pg/ml, 21.1 pg/ml, 158.5 × 10^9^/l and 20.0 ng/ml, respectively. Analysis of all dialysis-induced MCD cases revealed that the area under the ROC curve for α-klotho, FGF-23, IL-6, TNF-α, PLT count and BDNF were 0.58, 0.56, 0.71, 0.79, 0.72 and 0.62, respectively (Fig 5). Table 2 summarises the sensitivity, specificity, positive predictive value, negative predictive value and accuracy of α-klotho, FGF-23, IL-6, TNF-α, PLT count and BDNF for the diagnosis of dialysis-induced MCD.

**Table 2:** Evaluation of α-klotho, FGF-23, IL-6, TNF-α, PLT and BDNF as potential serum biomarkers for the diagnosis of hemodialysis-induced mild cognitive decline.

Evaluation index	cut-off value	Sensitivity	Specificity	Positive predictive value	Negative predictive value	Accuracy
α-klotho, (n)	469.5 pg/ml	41.4% (12/29)	44.8% (13/29)	42.9% (12/28)	43.3% (13/30)	43.1% (25/58)
FGF-23, (n)	1264.5 ng/ml	62.1% (18/29)	31.0% (9/29)	64.3% (18/28)	30.0% (9/30)	46.6% (27/58)
IL-6, (n)	1.8 pg/ml	17.2% (5/29)	44.8% (13/29)	17.9% (5/28)	43.3% (13/30)	31.0% (18/58)
TNF-α, (n)	21.1 pg/ml	37.9% (11/29)	10.3% (3/29)	39.3% (11/28)	10.0% (3/30)	24.1% (14/58)
PLT, (n)	158.5 × 10^9/L^	51.7% (15/29)	17.2% (5/29)	38.5% (15/39)	26.3% (5/19)	35.0% (20/58)
BDNF, (n)	20.0 ng/ml	13.8 (4/29)	65.5 (19/29)	28.6 (4/14)	43.2 (19/44)	40.0 (23/58)

BDNF: brain-derived neurotrophic factor; FGF-23: fibroblast growth factor-23; IL-6: interleukin-6; MCD: mild cognitive decline; PLT: platelet; ROC: receiver operating characteristic; TNF-α: tumour necrosis factor-α.

## Discussion

Hemodialysis is of critical importance to patients with CKD^15^. However, there is limited information on its potential deleterious effects on cognitive function. In the present study, we found that hemodialysis-related MCD was likely associated with changes in levels of serum biomarkers including BDNF, TNF-α and IL-6 as well as blood PLT counts. We did not find, however, any differences in serum levels of α-klotho or FGF-23 between the NCF and MCF groups.

BDNF is a protein that synthesised and widely distributed in the central nervous system (CNS) which plays an important role in the survival, differentiation and growth of neurons during CNS development^16,17^. Our and other studies suggest that patients with BDNF deficit are prone to neurological disorders and that clinical symptoms are significantly improved after amelioration of BDNF loss^18,19^. In the present study, we found that the NCF and MCD groups exhibited significantly decreased serum BDNF levels. Furthermore, a significant difference in serum BDNF levels observed between the MCD and NCF groups lend support that hemodialysis-related cognitive decline is associated with BDNF loss, consistent with a previous clinical study by Zoladz *et al*.^20^. Although the correlation between BDNF and MMSE score was not significant (*p* = 0.06), serum BDNF level was likely associated with the MoCA score. Furthermore, the ROC curve analysis revealed BDNF as a potential biomarker for the diagnosis of hemodialysis-induced MCD. In contrast, Shin *et al*.^21^ demonstrated that plasma BDNF level was significantly higher in hemodialysis patients than that of HC individuals. The reasons underlying this discrepancy, however, are not yet clear, and further studies on the role of BDNF in hemodialysis-related cognitive decline are required.

Alpha-klotho and FGF-23 have been widely reported to play important roles in a number of complications caused by hemodialysis, particularly in disorders of calcium and phosphorus metabolism^22,23^. Alpha-klotho, an anti-ageing protein, has been widely reported to be down-regulated in Alzheimer’s disease, an ageing-related disease characterized by cognitive decline^24,25^. Conversely, therapeutic strategies aimed at increasing CNS levels of α-klotho might reverse neurodegenerative processes^26^. However, our results failed to show a significant alteration in serum α-klotho levels in MCD patients compared with the NCF patients, although α-klotho was significantly decreased in CKD patients after hemodialysis. Furthermore, we previously reported the correlation between serum FGF-23 and Pi levels and that hemodiafiltration might be an effective method for clearing serum FGF-23 in hemodialysis patients with hyperphosphatemia^27^. FGF-23 was recently shown to not only play an important role in the molecular mechanisms underlying CKD and hemodialysis^28,29^ but also exert physiological functions in neuronal morphology and synaptic density^30^. In the present study, changes in FGF-23 levels were comparable to those of α-klotho, and no differences were observed between patients with cognitive decline and those with no decline. However, Drew *et al*.^14^ demonstrated that FGF-23 was negatively correlated with cognitive scores. They showed that FGF-23 was significantly up-regulated in patients with cognitive impairment. The discrepancies between their findings and our results might be explained by two reasons. First, patient enrollment criteria of the two studies are distinct. While Drew *et al*.^14^ included hemodialysis patients with cognitive impairment, the current study investigated hemodialysis patients with the milder form of cognitive decline. Second, detection methods used for biomarkers are different. While Drew *et al*.^14^ measured the C-terminal fragment of FGF-23, the assay we used measured the full-length FGF-23. Based on our findings, we suggest that hemodialysis can cause abnormal expression of serum FGF-23 and α-klotho but that hemodialysis-related MCD might not be associated with the changes in levels of FGF-23 and α-klotho.

Inflammatory cytokines play key roles in the pathogenesis and therapeutic mechanisms of CKD, hemodialysis-related side effects and brain diseases^31–33^. Montinaro *et al*.^13^ suggested that an increase in IL-6, an inflammatory cytokine, was linked to the presence of emotional symptoms. Furthermore, cognitive behavioural therapy significantly decreased the levels of inflammatory cytokines such as high-sensitivity C-reactive protein and IL-18 in 72 hemodialysis patients with sleep disorders^34^. In the present study, we demonstrated that TNF-α and IL-6 were significantly increased in hemodialysis patients and that their levels were significant different between the NCF and MCD groups. Moreover, MoCA but not MMSE was negatively correlated with serum levels of TNF-α and IL-6. This discrepancy might be explained by several factors. First, the MoCA has a wider range of measurements to assess cognitive function compared with the MMSE. The MMSE is suitable only for the detection of cognitive decline with impaired memory and language, and memory and language function account for only 60% of the cognitive function^35^. Second, the MoCA is more sensitive than the MMSE for assessing cognitive function. The MoCA is more likely to detect early-stage MCD and is more suitable for population screening^36^. Third, the MoCA is more rational in design and superior for the diagnosis of MCD. MCD is mainly characterised by delayed memory decline, which is detected more accurately with the MoCA^37^. Therefore, these findings demonstrate that hemodialysis-related MCD is accompanied by changes in serum levels of TNF-α and IL-6. However, the correlation and ROC analyses indicated that these two biomarkers might not be well suited for the diagnosis of MCD in CKD patients undergoing hemodialysis. Additional, detailed studies on the specific role of inflammatory cytokines in the pathogenesis of hemodialysis-related MCD are thus necessary.

PLTs are bioactive small pieces of cytoplasm that break off from the cytoplasmic lysis of mature bone marrow megakaryocytes^38^. Both mean PLT volume and PLT distribution width were reported to be decreased in patients with mild cognitive impairment and Alzheimer’s disease^39^. In addition, PLTs were previously shown to be exhausted after hemodialysis because of repeated hemodialysis stimulation and recurrent release of PLT degranulation products^40^. The results of the current study showed that blood PLT counts were significantly decreased in hemodialysis patients with MCD than that of healthy control individuals and hemodialysis patients with NCF. Furthermore, PLT counts were positively correlated with the MoCA and MMSE scores. Interestingly, substantial amounts of BDNF are expressed in PLTs, which contain 50- to 100-fold higher BDNF levels compared with the brain, and BDNF is released after PLT activation^31–43^. It is therefore likely that BDNF levels are closely related to PLT counts and function. The findings of the current study implicate both BDNF levels and PLT counts in hemodialysis-related MCD, suggesting that serum BDNF and PLT counts should be considered as objective indicators to aid in the diagnosis of hemodialysis-related MCD.

In conclusion, the present study revealed that abnormalities in serum levels of BDNF, TNF-α and IL-6 as well as PLT counts, but not α-klotho or FGF-23, might be contributing to hemodialysis-related MCD and that serum BDNF level and PLT counts might act as biomarkers for the diagnosis of hemodialysis-related MCD. Further detailed studies on the role of BDNF and PLTs in hemodialysis and its adverse effects are necessary.

## Methods

### Participants and study design

This prospective cohort study included 58 CKD patients undergoing hemodialysis between February and May 2017 at the Blood Purification Centre, The Third Affiliated Hospital of Soochow University. The study cohort was divided into the MCD and NCF groups, each comprising 29 patients, based on cognition tests, and 20 healthy individuals were also enrolled in the HC group. The MCD group included 15 males and 14 females, with an average age of 48.00 ± 7.20 years. In this group, primary diseases were chronic glomerulonephritis, type II diabetes mellitus, type I diabetes mellitus, benign arteriolar nephrosclerosis, polycystic kidney disease, IgA nephropathy and medullary sponge kidney in 15, 4, 1, 1, 1, 1 and 1 patient, respectively, whereas the cause of CKD was unknown in 5 patients. Similarly, 15 males and 14 females were enrolled in the NCF group, with an average age of 43.10 ± 7.80 years. In the NCF group, causes of CKD were chronic glomerulonephritis, nephrotic syndrome, type II diabetes mellitus, polycystic kidney disease, systemic lupus erythematosus and unknown in 17, 3, 1, 1, 1 and 6 patients, respectively. Eleven males and 9 females, with an average age of 45.00 ± 8.90 years, were included in the HC group.

Exclusion criteria were history of surgery for invasive intracranial pressure monitoring; mental or neurological diseases such as dementia, Alzheimer’s disease and schizophrenia; brain trauma; use of antiepileptic drugs, alcohol or illicit drugs. Written informed consent was obtained from all participants, and the study was approved by the Ethics Commission of Soochow University (Changzhou, China) and conducted in accordance with the principles of Declaration of Helsinki. This clinical study was registered with the Chinese Clinical Trial Register (Clinical Trial Number: ChiCTR-ROC-16009283).

### Treatment

All patients enrolled in this study underwent blood purification three times a week. Treatment regimen was hemodialysis twice a week combined with haemodiafiltration once a week or hemodialysis once a week combined with haemodiafiltration twice week. Hemodialysis was performed using the Behrin Low-Flux Poly (B. Braun Diacap LOPS15, Germany), and the haemodiafiltration was treated by a Braun high-throughput polysulfone membrane dialyser (B. Braun Diacap HIPS15). Each session was about four hours, and low-molecular-weight heparin was used for anticoagulation. Dialysis solutions were bicarbonate with an average blood flow velocity of 200–280 ml/min, and dialysis flow rate was 500 ml/min. Displacement volume using post-replacement was calculated by approximately 30% of ultrafiltration flow rate.

### Cognitive function tests

MMSE is a widely used cognitive screening test. Items in the MMSE address orientation, memory, recall, attention and naming objects, following verbal and written commands, writing a sentence and copying a figure, to reach a score range from 0 to 30. An MMSE score <27 is considered to indicate MCD.

MoCA was a recently developed test to address the shortcomings of MMSE in detecting mild cognitive impairment. MoCA has additional, more complex tasks including executive function. Items address orientation, drawing figures, processing speed, naming objects, memory, recall, attention, vigilance, repetition, verbal fluency and abstraction. MoCA score range is the same as that of MMSE (0–30). A MoCA score ≥26 is considered to indicate normal cognition. Additionally, MoCA adds one point for those whose educational level is 12 or fewer years.

### Laboratory tests

Routine blood tests were measured using an automated five-class blood cell analyser electrical impedance device (Sysmex Corporation, Japan). Blood urea nitrogen, creatinine, calcium, phosphorus and albumin were detected by an automated biochemical analyser (Leadman Biochemical, China). Parathyroid hormone measurement was achieved using chemiluminescence (Beckman, US), whereas serum α-klotho levels were measured using an enzyme-linked immunosorbent assay (Immuno-Biological Laboratories, Japan). Serum FGF-23, IL-6 and TNF-α levels were measured by a Luminex 200 protein liquid chip platform (Millipore, US) according to the manufacturer’s instructions.

### Statistical analysis

Data were expressed as means ± standard error of the mean (S.E.M.). Statistical analyses were performed using SPSS 13.0 (SPSS, Chicago, IL, USA). Comparisons between groups were performed by one-way analysis of variance followed by *post hoc* Tukey test, Student’s *t* test or χ^2^ test. Correlation analysis was conducted using Pearson’s product-moment coefficient. The diagnostic cut-off values, sensitivity, specificity and accuracy were determined by ROC curve analysis. A *p* value < 0.05 was considered to indicate a statistically significant difference.

## References

[1] Komenda P (2014). Cost-effectiveness of primary screening for CKD: a systematic review. Am. J. Kidney Dis..

[2] Weiner DE, McClean MD, Kaufman JS, Brooks DR (2013). The Central American epidemic of CKD. Clin. J. Am. Soc. Nephrol..

[3] Kraut JA, Madias NE (2016). Metabolic acidosis of CKD: an update. Am. J. Kidney Dis..

[4] Liu ZH (2013). Nephrology in China. Nat. Rev. Nephrol..

[5] Chen H, Iinuma M, Onozuka M, Kubo KY (2015). Chewing maintains hippocampus-dependent cognitive function. Int. J. Med. Sci..

[6] Krinsky-McHale SJ, Silverman W (2013). Dementia and mild cognitive impairment in adults with intellectual disability: issues of diagnosis. Dev. Disabil. Res. Rev..

[7] Tholen S (2014). Variability of cognitive performance during hemodialysis: standardization of cognitive assessment. Dement. Geriatr. Cogn. Dis..

[8] Drew DA (2015). Cognitive function and all-cause mortality in maintenance hemodialysis patients. Am. J. Kidney Dis..

[9] Kalaitzidis RG (2013). Risk factors for cognitive dysfunction in CKD and hypertensive subjects. Int. Urol. Nephrol..

[10] Hermann DM, Kribben A, Bruck H (2014). Cognitive impairment in chronic kidney disease: clinical findings, risk factors and consequences for patient care. J. Neural. Transm. (Vienna)..

[11] Fadili W (2014). Prevalence and risk factors of cognitive dysfunction in chronic hemodialysis patients. Aging Ment. Health.

[12] Slinin Y (2015). Timing of dialysis initiation, duration and frequency of hemodialysis sessions, and membrane flux: a systematic review for a KDOQI clinical practice guideline. Am. J. Kidney Dis..

[13] Montinaro V (2010). Emotional symptoms, quality of life and cytokine profile in hemodialysis patients. Clin. Nephrol..

[14] Drew DA (2014). FGF-23 and cognitive performance in hemodialysis patients. Hemodial. Int..

[15] KDOQI Clinical Practice Guideline for Hemodialysis Adequacy: 2015 update. *Am*. *J*. *Kidney Dis*. **66**, 884–930, 10.1053/j.ajkd.2015.07.015 (2015). 10.1053/j.ajkd.2015.07.015 26498416

[16] Lu B, Nagappan G, Lu Y (2014). BDNF and synaptic plasticity, cognitive function, and dysfunction. Handb. Exp. Pharmacol..

[17] Leal G, Comprido D, Duarte CB (2014). BDNF-induced local protein synthesis and synaptic plasticity. Neuropharmacology.

[18] Yang C, Shirayama Y, Zhang JC, Ren Q, Hashimoto K (2015). Regional differences in brain-derived neurotrophic factor levels and dendritic spine density confer resilience to inescapable stress. Int. J. Neuropsychopharmacol..

[19] Siegel PD, Bozelka BE, Reynolds C, George WJ (1989). Phase-dependent response of the lung to NO2 irritant insult. J. Environ. Pathol. Toxicol. Oncol..

[20] Zoladz JA (2012). Hemodialysis decreases serum brain-derived neurotrophic factor concentration in humans. Neurochem. Res..

[21] Shin SJ, Yoon HE, Chung S, Kim YG, Kim DJ (2012). Plasma brain-derived neurotrophic factor in hemodialysis patients. Int. J. Med. Sci..

[22] Suzuki K (1990). Hypoplastic left heart syndrome with premature closure of foramen ovale: report of an unusual type of totally anomalous pulmonary venous return. Heart Vessels.

[23] Otani-Takei N (2015). Association between serum soluble klotho levels and mortality in chronic hemodialysis patients. Int. J. Endocrinol..

[24] Dubal DB (2014). Life extension factor klotho enhances cognition. Cell Rep..

[25] Semba RD (2014). Klotho in the cerebrospinal fluid of adults with and without Alzheimer’s disease. Neurosci. Lett..

[26] Abraham CR, Chen C, Cuny GD, Glicksman MA, Zeldich E (2012). Small-molecule Klotho enhancers as novel treatment of neurodegeneration. ‎Future Med. Chem..

[27] Miao LY (2014). Effects of three blood purification methods on serum fibroblast growth factor-23 clearance in patients with hyperphosphatemia undergoing maintenance hemodialysis. Exp. Ther. Med..

[28] Lima F (2014). FGF-23 serum levels and bone histomorphometric results in adult patients with chronic kidney disease on dialysis. Clin. Nephrol..

[29] John GB, Cheng CY, Kuro-o M (2011). Role of Klotho in aging, phosphate metabolism, and CKD. Am. J. Kidney Dis..

[30] Hensel N (2016). Fibroblast growth factor 23 signaling in hippocampal cells: impact on neuronal morphology and synaptic density. J. Neurochem..

[31] Viana JL (2014). Evidence for anti-inflammatory effects of exercise in CKD. J. Am. Soc. Nephrol..

[32] Carlsson AC (2015). Endostatin, Cathepsin S, and Cathepsin L, and their association with inflammatory markers and mortality in patients undergoing hemodialysis. Blood Purif..

[33] Norden DM, Godbout JP (2013). Review: microglia of the aged brain: primed to be activated and resistant to regulation. Neuropathol. Appl. Neurobiol..

[34] Chen HY (2011). Cognitive-behavioral therapy for sleep disturbance decreases inflammatory cytokines and oxidative stress in hemodialysis patients. Kidney Int..

[35] Lee JY (2008). Brief screening for mild cognitive impairment in elderly outpatient clinic: validation of the Korean version of the Montreal Cognitive Assessment. J. Geriatr. Psychiatry. Neurol..

[36] Smith T, Gildeh N, Holmes C (2007). The Montreal Cognitive Assessment: validity and utility in a memory clinic setting. Can. J. Psychiatry..

[37] Oudman E (2014). The Montreal Cognitive Assessment (MoCA) is superior to the Mini Mental State Examination (MMSE) in detection of Korsakoff’s syndrome. Clin. Neuropsychol..

[38] Grozovsky R, Giannini S, Falet H, Hoffmeister KM (2015). Regulating billions of blood platelets: glycans and beyond. Blood.

[39] Wang RT, Jin D, Li Y, Liang QC (2013). Decreased mean platelet volume and platelet distribution width are associated with mild cognitive impairment and Alzheimer’s disease. J. Psychiatry. Res..

[40] Mekawy MA, Habashy DM, Abd El-Mohsen WA (2015). Effect of hemodialysis on platelet function in end-stage renal disease Egyptian patients using *in vitro* closure time test (PFA-100 analyzer). Platelets.

[41] Burnouf T, Kuo YP, Blum D, Burnouf S, Su CY (2012). Human platelet concentrates: a source of solvent/detergent-treated highly enriched brain-derived neurotrophic factor. Transfusion.

[42] Turck P, Frizzo ME (2015). Riluzole stimulates BDNF release from human platelets. BioMed Res. Int..

[43] Tamura S (2011). Release reaction of brain-derived neurotrophic factor (BDNF) through PAR1 activation and its two distinct pools in human platelets. Thromb. Res..

